# Assessment of the quality of recommendations from 161 clinical practice guidelines using the Appraisal of Guidelines for Research and Evaluation–Recommendations Excellence (AGREE-REX) instrument shows there is room for improvement

**DOI:** 10.1186/s13012-020-01036-5

**Published:** 2020-09-18

**Authors:** Ivan D. Florez, Melissa C. Brouwers, Kate Kerkvliet, Karen Spithoff, Pablo Alonso-Coello, Jako Burgers, Francoise Cluzeau, Beatrice Férvers, Ian Graham, Jeremy Grimshaw, Steven Hanna, Monika Kastner, Michelle Kho, Amir Qaseem, Sharon Straus

**Affiliations:** 1 Department of Pediatrics, Universidad de Antioquia, Calle 67 # 53-108, 050001 Medellin, Colombia; 2 Department of Oncology, McMaster University, Juravinski Site, G2 Wing, 1280 Main Street West, Hamilton, Ontario L8S 4L8 Canada; 3 School of Epidemiology and Public Health, University of Ottawa, 101F, 600 Peter Morand, Ottawa, Ontario K1G 5Z3 Canada; 4 Iberoamerican Cochrane Centre, Hospital de la Santa Creu i Sant Pau, C/Sant Antoni M. Claret 167, Pavelló 18 planta 0, 08025 Barcelona, Spain; 5 Dutch College of General Practitioners, PO Box 3231, Utrecht, The Netherlands; 6 Imperial College London, St. Mary’s Hospital (Room 1070, Queen Elizabeth the Mother Wing), Praed Street, London, W2 1NY UK; 7 Département Cancer et Environnement, Centre Léon Bérard, 28 rue Laënnec, 69373, Cedex 08 Lyon, France; 8 Ottawa Hospital Research Institute, University of Ottawa, 501 Smyth Road, Box 711, Ottawa, Ontario K1H 8L6 Canada; 9 Faculty of Health Sciences, McMaster University, 2C Health Sciences Centre, 1280 Main Street West, Hamilton, Ontario L8S 4L8 Canada; 10 North York General Hospital, 4001 Leslie Street, Toronto, Ontario M2K 1E1 Canada; 11 Institute of Applied Health Sciences McMaster University, 1280 Main Street West, Hamilton, Ontario L8S 4L8 Canada; 12 American College of Physicians, 190 N Independence Mall West, Philadelphia, PA 19106-1572 USA; 13 Li Ka Shing Knowledge Institute of St. Michael’s Hospital, 30 Bond Street, Shuter 2-026, Toronto, Ontario M5B 1W8 Canada

**Keywords:** Clinical practice guidelines, Practice guidelines, Recommendations, Quality of health care, AGREE tool, Implementability

## Abstract

**Objective:**

To assess the quality of recommendations from 161 clinical practice guidelines (CPGs) using AGREE-REX-D (Appraisal of Guidelines REsearch and Evaluation-Recommendations Excellence Draft).

**Design:**

Cross-sectional study

**Setting:**

International CPG community.

**Participants:**

Three hundred twenty-two international CPG developers, users, and researchers.

**Intervention:**

Participants were assigned to appraise one of 161 CPGs selected for the study using the AGREE-REX-D tool

**Main outcome measures:**

AGREE-REX-D scores of 161 CPGs (7-point scale, maximum 7).

**Results:**

Recommendations from 161 CPGs were appraised by 322 participants using the AGREE-REX-D. CPGs were developed by 67 different organizations. The total overall average score of the CPG recommendations was 4.23 (standard deviation (SD) = 1.14). AGREE-REX-D items that scored the highest were (mean; SD): *evidence* (5.51; 1.14), *clinical relevance* (5.95; SD 0.8), and *patients/population relevance* (4.87; SD 1.33), while the lowest scores were observed for the *policy values* (3.44; SD 1.53), *local applicability* (3,56; SD 1.47), and *resources, tools, and capacity* (3.49; SD 1.44) items. CPGs developed by government-supported organizations and developed in the UK and Canada had significantly higher recommendation quality scores with the AGREE-REX-D tool (*p* < 0.05) than their comparators.

**Conclusions:**

We found that there is significant room for improvement of some CPGs such as the considerations of patient/population values, policy values, local applicability and resources, tools, and capacity. These findings may be considered a baseline upon which to measure future improvements in the quality of CPGs.

Contributions to the literature

- We applied the AGREE II and the recently developed tool (AGREE-REX draft version), to assess quality, credibility, and implementability of 161 international clinical practice guidelines (CPGs). The AGREE REX draft tool was applied by 322 guidelines’ developers, users and researchers from 51 countries.
-The scores of the AGREE REX draft tool items were higher in those items related to the quality of the evidence and the clinical relevance. The items related to patients and population relevance and implementation relevance scored in the mid-range, while the items related to patients/population or policy values, the alignment of values, the local applicability, and the resouces, tools, and capacity items scored low.
- CPGs produced by government-supported organizations scored higher on all the items of the AGREE-REX draft tool than those produced by professional societies or other types of groups, and CPGs produced in UK and Canada scored higher in selected items in comparison to USA and international CPGs
- The correlations between the overall AGREE-REX draft tool and AGREE II domains were low, except for the applicability domain where the correlation was modest.

## Introduction

Clinical practice guidelines (CPGs) are systematically developed statements informed by a systematic review of evidence and an assessment of the benefits and harms of alternative care options with the aim of optimizing patient care [1–3]. However, concerns about variation in the quality of CPGs and their resultant recommendations exist in the literature [1, 3, 4]. The AGREE II is an established instrument, used internationally, to evaluate the overall methodological quality of CPGs and to serve as a methodological blueprint to inform CPG development and reporting [5–7]. The AGREE II focuses on the entire CPG *development process*. As its complement, the AGREE-REX (Appraisal of Guidelines REsearch and Evaluation-Recommendations EXcellence) was designed to focus specifically on CPG *recommendations* and the justifications that underpin them [8]. Its development was in response to data demonstrating high-quality CPG processes, although necessary, are not always sufficient to yield individual CPG recommendations that are clinically credible and implementable [9, 10].

The prototype of the AGREE-REX (the AGREE-REX-D) and the AGREE II was applied to 161 guidelines. In this article, we present the results of this assessment, identify areas for CPG recommendation improvement, and compare the evaluative information garnered by both tools.

## Materials and methods

This study represents a component of a larger program of research designed to create the AGREE-REX version 1 (AGREE-REX-D); the technical components of this program of research are reported elsewhere [8]. Our main study was designed to create the AGREE REX tool following a mixed-methods project, and this manuscript presents the cross-sectional study that summarizes the assessment of the selected CPGs during the development of the AGREE REX-D). This study received ethics approval from the Hamilton Integrated Research Ethics Board (project #13-700).

### Participants

Participants included CPG developers, clinicians, implementers, and other users. They were purposefully recruited through a variety of channels including social media and CPG organizations, such as the Guidelines International Network (G-I-N), G-I-N North America regional community, Knowledge Translation (KT) Canada, Canadian Agency for Drugs and Technologies in Health (CADTH), Canadian Partnership Against Cancer, Cancer Care Ontario, and to investigators known in the CPG research community. The study was also advertised on the AGREE social media accounts (Facebook and Twitter), and My AGREE PLUS (online platform for appraising CPGs with the AGREE II tool, www.agreetrust.org) registered users were invited to participate.

### CPGs

CPGs in multiple clinical specialty areas were collected from the Agency for Healthcare Research and Quality (AHRQ) National Guidelines Clearinghouse database [11]. Using the database’s advanced search function, we identified CPGs that were (1) published between 2013 and 2015; (2) written in English language, and (3) no more than 50 pages in length for the CPG core document. The resulting list of CPGs was reviewed and the following were excluded: guidelines addressing organizational rather than clinical topics, technology assessments; CPGs not available for free to the public; and CPGs for which the link in the database were not functional. Descriptive information was extracted from each CPG, including type of authoring organization (government supported vs. professional society vs other/not clear), disease topic (cancer vs. non-cancer), and country of authoring group (USA, UK, Canada, or international).

### Procedure

Participants received individualized password-protected access to the study materials, which included links to a downloadable PDF format of the AGREE-REX-D, the CPG to which they were randomly assigned, and the online survey platform (LimeSurvey) to record their scores. Participants were asked to review the AGREE-REX-D manual and items, read the CPG, and then evaluate it by applying the tool and recording their item ratings in LimeSurvey. Participants were provided with no formal training or orientation of the tool by members of the team. The AGREE-REX-D manual provided definitions of the items and instructions on how to assess and score them. An email reminder was sent at 2 weeks from the participant’s initial start date informing them of their deadline in 1 week. Deadline extensions were given when requested. Evaluations were completed between May 2016 and March 2017. Participants were offered a $50 CAD pre-paid virtual gift card for completing the study. All communication with participants was done by the staff of AGREE Scientific office.

### Outcomes

#### AGREE-RE-DX scores

The prototype of AGREE-REX-D comprised 11 items within 4 themes (Table 1). Each item was rated using a 7-point scale applied to two quality attributes, with higher scores reflecting higher quality. The two attributes were the following:

- Extent to which quality features were documented in the CPG
- Extent to which quality features were considered in formulating the recommendations.

**Table 1:** AGREE-REX

AGREE-REX domains and items	
Domains	Items
1. Evidence justification	1. Evidence
2. Clinical applicability justification	2. Clinical relevance 3. Relevance to patients/populations 4. Implementation relevance
3. Values justification	5. Guideline developer values 6. Target user values 7. Patient population values 8. Policy values 9. Alignment of values
4. Feasibility considerations	10. Local applicability 11. Resources, capacity, and tools

The instrument concludes with two general quality assessments: overall credibility and overall implementability of the CPG recommendations.

### AGREE II evaluations

For exploratory purposes, the CPGs were also assessed, independently, using the AGREE II by two members of the AGREE Scientific team. The AGREE II includes 23 items within 6 domains and 2 overall assessments [5]. The 23 items are assessed with a 7-point scale (1 = strongly disagree; 7 = strongly agree), with high scores reflecting more favorable quality results. Discrepancies in scoring were resolved by consensus when required.

### Scoring

For each CPG, an AGREE-REX-D item score was derived for each of the 11 items by averaging scores on the 7-point scale between the two raters. A mean overall AGREE-REX-D score was calculated for each CPG by averaging across the 11 items. Finally, mean scores for overall credibility and overall implementability items were derived by averaging scores between the two raters.

AGREE II tool mean domain scores were derived by summing the scores across the two appraisers and standardizing them as a percentage of the maximum possible score a CPG could achieve for that domain [5]. Before these scores were summed and calculated, the independent appraisers were required to reach a consensus on any AGREE II item scores that were two or more points apart on the 7-point scale.

### Sample size calculation

The sample size calculation was based on a separate methodological goal to conduct a reliability study of the AGREE-REX-D tool based on the interrater reliability outcome. Based on consensus by the team, we made the following assumptions: two raters per CPG, an intraclass correlation coefficient of 0.6, and a confidence interval from 0.5 to 0.7. We determined that we required 316 participants to appraise 158 CPGs: each participatant rated one CPG using the AGREE-REX-D and each CPG was rated by two independent raters. Additional information on the details of the sample size calculation can be found elsewhere [8]

### Analytical framework

Descriptive measurements were used to summarize the AGREE-REX item and overall scores. A series of one-way ANOVA tests was used to examine mean differences in the AGREE-REX-D item scores and the overall score as a function of the following characteristics: type of authoring organization (government-supported vs. professional societies vs. other), disease topic (cancer vs. not cancer), and country of development (USA vs. UK vs. Canada vs. international). International guidelines category included guidelines co-developed by two or more countries or developed by international organizations or societies. Descriptive measures were used to summarize AGREE II domain scores. Finally, correlations between mean overall AGREE-REX-D scores and AGREE II domain scores were calculated. Analyses were performed using Stata 15.0 (StataCorp. 2017. *Stata Statistical Software: Release 15*. College Station, TX: StataCorp LLC).

## Results

### Participants

Descriptive statistics of the participants are listed in Tables 2 and 3.

**Table 2:** Participants demographics (*n* = 322)

Demographic characteristics	Frequency ***n*** (%)
**Sex**	
Female	202 (62.5)
Male	115 (35.7)
I prefer not to disclose	5 (1.6)
**Age**	
19 or younger	2 (0.6)
20–29	49 (15.2)
30–39	100 (31.1)
40–49	83 (25.8)
50–59	63 (19.6)
60–69	23 (7.1)
70 and older	2 (0.6)
**Experience with AGREE II**	
No experience	70 (21.7)
Some experience	122 (37.9)
Experienced	88 (27.3)
Very experienced	42 (13)
**Geographic location**	
North America	177 (55)
Europe	76 (23.6)
Asia	24 (7.5)
South America	32 (9.9)
Africa	7 (2.2)
Oceania	6 (1.9)
**First language**	
English	188 (58.4)
Spanish	51 (15.8)
Italian	14 (4.3)
Chinese	13 (4)
Dutch	10 (3.1)
Portuguese	7 (2.2)
French	4 (1.2)
Greek	3 (0.9)
Ukrainian	3 (0.9)
Other	29 (9)
**Participants’ roles with clinical practice guidelines (PG)**^a^	
CPG developer—clinical expert	85 (26.4)
CPG developer—patient/public representative	15 (4.7)
CPG developer—methodologist	170 (52.8)
CPG user—health care provider	102 (31.7)
CPG user—administrator/policy maker/manager	38 (11.8)
CPG user—patient/member of the public	20 (6.2)
Researcher	159 (49.4)
Other (e.g., librarian, student)	25 (7.8)

^a^Participants could select more than one role

**Table 3:** Comparison of mean overall AGREE-REX scores by participant demographic feature

Participant demographic	*t* test statistic^a^	*p* value
Experience vs. no experience	4.04	.001
North America vs. all other regions	2.86	.004
English vs. non-English	1.056	.290
PG developers Vs. PG users and researchers	− 2.29	.023

^a^Equal variances assumed

### CPGs

We appraised 161 CPGs. The CPGs targeted a range of diseases and clinical problems including cancer, infectious diseases, pregnancy and child birth, mental health, nervous system disorders, respiratory, digestive, genitourinary, blood and endocrine disorders, and musculoskeletal, among others. With the exception of cancer (*n* = 38), the number of CPGs for each unique disease was small (< 8) making other comparisons by disease topic not viable. CPGs were developed by 67 different international organizations (see Additional file 1 Appendix 1). Organizations that produced the CPGs were government-supported in less than a third of cases (*n* = 46; 28.6%), and they were authored by groups most often located in USA (*n* = 89; 55.3%) or the UK (*n* = 46; 28.6%). CPGs were all published between 2013 and 2015. The list of appraised CPGs can be accessed in the supplementary file.

### AGREE-REX (see Table 4)

#### AGREE-REX.D performance for all CPGs

The mean overall AGREE-REX score across the 161 CPGs was 4.23 (SD 1.14). There was variability in performance across the individual 11 items, with 6 that scored above the middle point of 4.0 on the response scale. The mean overall credibility and overall implementability assessments were 4.78 (SD 1.24) and 4.19 (SD 1.23), respectively.

**Table 4:** ^a^AGREE-REX Scores For Specific Items, Overall Items (across the 11 Items), and Overall Assessments

Practice Guidelines (PG) Features	Specific Items Means											Overall Means		Overall Assessments		# PGs
	Evidence	Clinical Relevance	Patient/pop. Relevance	Implement-ation Relevance	Values Developers	Values Users	Values Patient/Pop	Values Policy	Alignment of Values	Local Applicability	Resources, Tools, Capacity	Mean Overall Score	% Score Mean	Clinical Credibility	Implement- ability	
**All Guidelines**	**5.15 (1.33)**	**5.47 (1.18)**	**4.87 (1.33)**	**4.46 (1.52)**	**4.61 (1.45)**	**4.28 (1.52)**	**3.85 (1.61)**	**3.44 (1.53)**	**3.42 (1.44)**	**3.56 (1.47)**	**3.49 (1.44)**	**4.23 (1.14)**	**54 (19)**	**4.78 (1.24)**	**4.19 (1.23)**	**161**
**Type of Organization**																
Government Supported	5.61 (1.14)	5.95 (0.8)	5.57 (0.98)	5.48 (0.89)	5.26 (1.2)	4.97 (1.14)	4.83 (1.34)	4.56 (1.15)	4.11 (1.32)	4.52 (1.3)	4.41 (1.11)	5.02 (0.77)	67 (13)	5.28 (0.96)	4.85 (1.07)	46
Professional Societies	4.98 (1.35)	5.24 (1.26)	4.57 (1.36)	4.04 (1.55)	4.30 (1.48)	3.98 (1.58)	3.41 (1.52)	2.99 (1.46)	3.1 (1.38)	3.17 (1.39)	3.06 (1.39)	3.89 (1.2)	48 (19)	4.56 (1.31)	3.89 (1.21)	109
Other or Not Clear	4.83 (1.63)	6.17 (0.68)	5.00 (1.41)	4.42 (1.24)	5.17 (0.93)	4.5 (1.38)	4.42 (2.04)	3.00 (0.89)	3.75 (1.63)	3.33 (0.82)	4.25 (1.29)	4.44 (0.99)	57 (17)	4.83 (0.82)	4.50 (0)	6
*p-value*	**0.021**	**<0.001**	**<0.001**	**<0.001**	**<0.001**	**<0.001**	**<0.001**	**<0.001**	**<0.001**	**<0.001**	**<0.001**	**<0.001**	**<0.001**	**0.004**	**<0.001**	
**Country**																
US	5.06 (1.39)	5.33 (1.28)	4.68 (1.4)	4.17 (1.58)	4.51 (1.49)	4.12 (1.62)	3.61 (1.66)	3.08 (1.47)	3.31 (1.54)	3.26 (1.47)	3.22 (1.47)	4.03 (1.21)	51 (20)	4.62 (1.31)	3.98 (1.26)	89
UK	5.26 (1.23)	5.74 (0.9)	5.22 (1.13)	5.04 (1.25)	4.86 (1.39)	4.76 (1.21)	4.23 (1.31)	4.16 (1.41)	3.66 (1.26)	4.18 (1.48)	4.12 (1.41)	4.66 (0.95)	61 (16)	5.06 (1.06)	4.51 (1.19)	46
Canada	5.46 (1.21)	5.87 (0.64)	5.17 (1.19)	4.75 (1.5)	4.67 (1.51)	4.5 (1.58)	4.46 (2)	3.87 (1.61)	3.79 (1.48)	3.83 (1.27)	3.42 (1)	4.53 (1.08)	59 (18)	4.92 (1.12)	4.50 (1.1)	12
International	5.18 (1.38)	5.18 (1.44)	4.64 (1.42)	4.18 (1.47)	4.39 (1.47)	3.54 (1.31)	3.64 (1.67)	2.96 (1.39)	2.96 (1.18)	3.18 (0.99)	3.25 (1.14)	3.91 (0.98)	49 (16)	4.75 (1.38)	4.21 (1.03)	14
*p-value*	0.7	0.1146	0.1192	**0.011**	0.5495	**0.025**	0.0932	**<0.001**	0.2609	**0.0036**	**0.0056**	**0.0106**	**0.0106**	0.2649	**0.0851**	

^a^Mean (SD); *US* United States, *UK* United Kingdom, *Implement* implementability, *Patient/Pop* Patients/population

#### AGREE-REX-D performance by type of organization

Statistically significant differences (i.e., *p* < 0.05) were found as a function of organization type for each of the mean AGREE-REX-D items, the mean overall AGREE-REX-D score, and the overall implementability and overall credibility assessments. In each case, more favorable ratings were found among CPGs produced by government-supported organizations. The item scores of CPGs produced by government-supported organizations (*n* = 46) ranged from 4.41 (SD 1.11) to 5.95 (SD 0.8); the scores of CPG produced by professional societies (*n* = 109) ranged from 2.99 (SD 1.46) to 5.24 (SD 1.26); and the scores of CPG produced by other types of organizations (*n* = 6), ranged from 3.00 (SD 0.89) to 6.17 (SD 0.68). Of note, in 5 of the 11 cases, the AGREE-REX-D item means across the organization types fell within the positive ends of the response scale (*m* ≥ 4) despite there being statistically significant differences between them. In contrast, in 6 of the 11 cases, the overall means of the AGREE-REX-D items straddled the mid-point of the scale—suggesting some organizations tended to perform lower than the mid-point and others perform higher than the mid-point of the scale.

#### AGREE-REX-D performance by country of CPG authoring group

The country of the authoring CPG organization showed differences in AGREE-REX quality scores as well. Statistically significant differences (i.e., *p* < 0.05) for five AGREE-REX items (implementation relevance, target user values, policy values, local applicability, and resources, tools, and capacity), and the mean overall AGREE-REX score were found. Differences as a function of authoring group approached, but did not reach, statistical significance for the overall implementability assessment. For each of these comparisons, the CPGs produced in the UK and Canada showed higher scores. The item scores of CPGs published from the UK ranged from 3.66 (SD 1.26) to 5.74 (SD 0.90); from Canada ranged from 3.42 (SD 1.0) to 5.87 (SD 0.64); from the USA ranged from 3.08 (SD 1.47) to 5.06 (SD 1.39); and from international organizations ranged from 2.96 (SD 1.39) to 5.18 (SD 1.44)). In all but one case, overall AGREE-REX-D item means straddled the mid-point of the scale where there was a significant difference between the groups.

#### AGREE-REX-D performance by disease

No significant differences emerged between cancer and non-cancer CPGs scores; this held true for each of the AGREE-REX items and the mean overall AGREE-REX-D score (*p* > 0.5; means not presented).

### AGREE II (see Table 5)

The AGREE II domain scores for the CPGs are displayed in the Table 5. *Scope and purpose*, and *clarity of presentation* were the domains with the highest scores, while the *applicability* domain had the lowest score.

**Table 5:** Average AGREE II Domain Scores (*n* = 161 PGs)

AGREE II Domains	Mean	SD	Min	Max
Scope and Purpose	75.3	14.4	33	100
Stakeholder Involvement	56.0	16.4	19	86
Rigour of Development	56.6	16.6	10	91
Clarity and Presentation	81.2	12.3	33	100
Applicability	36.5	21.4	0	94
Editorial Independence	57.4	24.9	0	100

### AGREE II and AGREE-REX

The correlations between the overall AGREE-REX-D and AGREE II domains were low (*r* < 0.30) except for the applicability domain where the correlation was modest at *r* = 0.38 [8]. Overall, AGREE-REX scores were higher among appraisers with no AGREE II experience compared to those with AGREE II experience.

## Discussion

We appraised 161 CPGs with the prototype of the AGREE-REX-D tool and the AGREE II tool. The most favorable AGREE-REX ratings (means > 5.0) were found for the *evidence* and *clinical relevance* items; ratings that fell in the more moderate range of the scale (means > 4.0 and < 5.0) were found for the *patient/population relevance*, *implementation relevance, developers’ values* and *users’ values* items; and least favorable ratings that fell below the mid-point of the scale (means < 4.0) were found for *patients/population values, policy values, alignment of values, local applicability* and *resources, tools and capacity* items. CPGs produced by government-supported organizations scored higher on all the items of the AGREE-REX-D than those produced by professional societies or other types of groups, and CPGs produced in UK and Canada scored higher in selected items in comparison to USA and international CPGs. The confidence intervals around the mean AGREE-REX scores were large.

The distribution of the mean scores across the 11 items is not surprising. CPG methods research has focused largely on issues directly relevant to creating the evidence base. As a consequence, some AGREE-REX concepts are easier to achieve success because there exists tools and resources to support thier operationalization (e.g., tools designed by the GRADE working group [12]). In contrast, resources to operaitonalize other concepts are more elusive. For example, continued methodological development is needed to adequately measure and report values across diverse stakeholder groups so that they are reliable, valid, and usable. Similarly, systematic strategies to incorporate these perspectives into the framing of recommendations are required [13].

As previously reported with the evaluation of the AGREE II [14], lower scores with some AGREE-REX-D items may reflect inadequate reporting and not poor quality in methodological execution [6]. Developers may have followed appropriate steps but not reported them in the CPG documentation and, as a consequence, could not be assessed. Also, it is possible that some conceptual elements reflected in the AGREE-REX-D (e.g., concepts related to implementation activities) are not the responsibility of the CPG developer directly, but perhaps by another party or group within their specific settings [12]. Thus, the AGREE-REX could provide a signal to individuals who are ultimately responsible for action about where gaps and barriers to this goal exist so that corrective action can be taken.

Differences in mean overall AGREE-REX-D scores as a function of the type of organization may reflect the greater interest or great capacity of government-supported organizations to seek out a broader range of values or invest in additional methodological steps that lead to higher quality scores than do other types of development groups. These data align with initial appraisal findings using the original AGREE instrument, in which CPGs developed by government-supported organizations also had the most favorable quality scores [15]. CPG panels with more resources (financial and access to skilled methodologists) confer quality benefits and setting quality standards too high may have the unintended consequence of increasing the disparities between the “have much” and “have less” jurdisdictions. Similar differences and similar concerns were raised in the assessment of CPGs with the original AGREE instrument [15].

Our study has several limitations. First, we only included English-language CPGs in the analysis. As a result, we have no data on the unique strengths or limitations related to credibility and implementability of non-English CPGs. This provides an opportunity for future research studies. Additionally, in order to optimize the feasibility of the study and candidates’ interests to participate, we only included CPGs that were less than 50 pages in length (excluding appendices and tables). Although the length of the CPG document is not necessarily associated with the quality, credibility and implementability, the restriction we imposed may have resulted in the exclusion of lengthy CPGs that may have more information and perhaps could have been scored higher. In addition, while 161 CPGs were evaluated, they were not from 161 unique developers. This could potentially be a source of confounding. Finally, the penultimate prototype of the AGREE-REX-D was used and not the final version. While there is considerable overlap between the two, future status reports must account for these differences when reflecting on changes in scores over time.

## Conclusion

As part of the development of the AGREE-REX tool, we assessed 161 CPG recommendations from different organizations around the world using the draft version of the tool. We found that there is significant room for improvement in some CPG recommendation elements. The most unfavorable ratings were found in the following items: patients/population values, policy values, alignment of values, local applicability and resources, tools and capacity. It should also be noted that statistically significant higher scores were found in guidelines developed by government-supported organizations (in comparison to those produced by professional or specialist societies or others), and in guidelines developed in the UK and Canada (in comparison to those produced in the USA and internationally.

Since the AGREE-REX can be used as a methodological blueprint to inform the development and reporting of high-quality recommendations, our findings may be used as a baseline upon which to measure future improvements in the quality of CPG recommendations.

## Supplementary information

**Additional file 1.** List of development organizations of clinical practice guidelines assessed.

## Data Availability

The analyses are available from the corresponding author.
