# Supplementary material for: Assessment of the quality of recommendations from 161 clinical practice guidelines using the Appraisal of Guidelines for Research and Evaluation–Recommendations Excellence (AGREE-REX) instrument shows there is room for improvement
Source: Implement Sci. 2020 Sep 18;15:79. doi: 10.1186/s13012-020-01036-5 (PMC7501649; doi:10.1186/s13012-020-01036-5)
Supplement: Supplementary file 1 — Additional file 1. List of development organizations of clinical practice guidelines assessed. [file 13012_2020_1036_MOESM1_ESM.docx]

**Appendix 1. List of Practice Guideline Development Organizations (***(In brackets, the number of guidelines included)*

1. American Academy of Child and Adolescent Psychiatry (2)
2. American Academy of Dermatology (1)
3. American Academy of Family Physicians (1)
4. American Academy of Neurology (2)
5. American Academy of Neurology; American Association of Neuromuscular and Electrodiagnostic Medicine (1)
6. American Academy of Otolaryngology - Head and Neck Surgery Foundation (5)
7. American Academy of Pediatrics (1)
8. American Association of Blood Banks (1)
9. American Association of Manipulation Under Anesthesia Providers (1)
10. American Association of Neurological Surgeons; Congress of Neurological Surgeons (1)
11. American Association of Neuroscience Nurses (2)
12. American College of Cardiology Foundation; American Heart Association (3)
13. American College of Cardiology Foundation; American Heart Association; The Obesity Society (1)
14. American College of Chest Physicians (1)
15. American College of Chest Physicians; Canadian Thoracic Society (1)
16. American College of Emergency Physicians (2)
17. American College of Gastroenterology (1)
18. American College of Medical Genetics and Genomics (1)
19. American College of Physicians (6)
20. American College of Surgeons (1)
21. American Dental Association Council on Scientific Affairs (1)
22. American Gastroenterological Association Institute (2)
23. American Heart Association; American Stroke Association (1)
24. American Pain Society; College on Problems of Drug Dependence (1)
25. American Society for Gastrointestinal Endoscopy (1)
26. American Society for Parenteral and Enteral Nutrition (1)
27. American Society for Radiation Oncology (2)
28. American Society of Anesthesiologists (1)
29. American Society of Clinical Oncology (9)
30. American Society of Clinical Oncology; Cancer Care Ontario (1)
31. American Thoracic Society (1)
32. American Thoracic Society; European Respiratory Society (1)
33. American Urological Association Education and Research, Inc (6)
34. British Association for Sexual Health and HIV (3)
35. British Association of Dermatologists (1)
36. British Committee for Standards in Haematology (7)
37. British Committee for Standards in Haematology; British Society of Blood and Marrow Transplantation (1)
38. Canadian Agency for Drugs and Technologies in Health (1)
39. Canadian Chiropractic Association; Canadian Federation of Chiropractic Regulatory and Educational Accrediting Boards (Federation) (1)
40. Canadian Hypertensive Disorders of Pregnancy Working Group (1)
41. Canadian Task Force on Preventive Health Care (3)
42. Cincinnati Children's Hospital Medical Center (2)
43. Congress of Neurological Surgeons (1)
44. Eastern Association for the Surgery of Trauma (4)
45. Endocrine Society (7)
46. European Academy of Allergy and Clinical Immunology (2)
47. European Academy of Allergy and Clinical Immunology Food Allergy and Anaphylaxis Guidelines Group (1)
48. European Society for Pediatric Gastroenterology, Hepatology, and Nutrition; North American Society for Pediatric Gastroenterology, Hepatology, and Nutrition (1)
49. Genetic Metabolic Dietitians International; Southeast Regional Newborn Screening and Genetics Consortium (1)
50. Institute for Clinical Systems Improvement (1)
51. International Lyme and Associated Diseases Society (1)
52. International Society for Oral Oncology; Multinational Association of Supportive Care in Cancer (1)
53. International Society for Heart and Lung Transplant/American Thoracic Society/European Respiratory Society Bronchiolitis Obliterans Syndrome Task Force Committee (1)
54. Joint National Committee (1)
55. Kidney Disease: Improving Global Outcomes (1)
56. New York State Department of Health (2)
57. National Institute for Health and Care Excellence (29)
58. Program in Evidence-based Care (1)
59. Royal College of Obstetricians and Gynaecologists (2)
60. Scottish Intercollegiate Guidelines Network (2)
61. Society of Obstetricians and Gynaecologists of Canada (5)
62. The Orthopaedic Section of the American Physical Therapy Association, Inc. (1)
63. U.S. Preventive Services Task Force (5)
64. Undersea & Hyperbaric Medical Society, Inc (1)
65. University of Michigan Health System (5)
66. World Gastroenterology Organisation (1)
67. World Health Organization (1)
